# Metal Ions in Biological Systems, Volume 33

**DOI:** 10.1155/MBD.1997.113

**Published:** 1997


### METAL IONS IN BIOLOGICAL SYSTEMS
### VOLUME 33

**Edited by A. Sigel and H. Sigel, Marcel Dekker, Inc. (1996)**
**712 pages, ISBN# : 0-8247-9688-8**

Chapter 1 : Molecular modeling of transition metal complexes with nucleic acids and their constituents, by J. Kozelka
Chapter 2 : Zinc complexes as targeting agents for nucleic acids, by E. Kimura and M. Shionoya
Chapter 3 : Metallocene interactions with DNA and DNA-processing enzymes, by L. Y. Kuo, A. H. Liu and T. J. Marks
Chapter 4 : Evidence for a catalytic activity of the DNA double helix in the reaction between DNA, platinum(II) and the intercalators, by M. Boudvillain, R. Dalbiès and M. Leng
Chapter 5 : *Trans*-diammineplatinum(II) : What makes it different from *cis*-DDP? Coordination chemistry of a neglected relative of cisplatin and its interaction with nucleic acids, by B. Lippert
Chapter 6 : Metal ions in multiple-stranded DNA, by M. Sabat and B. Lippert
Chapter 7 : DNA interactions with substitution-inert transition metal ion complexes, by B. Norden, P. Lincoln, B. Akerman and E. Tuite
Chapter 8 : Effect of metal ions on the fluorescence of dyes bound to DNA, by V. G. Bregadze, J. G. Chkhaberidze and I. G. Khutsishvili
Chapter 9 : Photolytic covalent binding of metal complexes to DNA, by M. A. Billadeau and H. Morrison
Chapter 10 : Electrochemically activated nucleic acid oxidation, by D. H. Johnston, T. W. Welch and H. H. Thorp
Chapter 11 : Electron transfer between metal complexes bound to DNA : Is DNA a wire?, by E. D. A. Stemp and J. K. Barton
Chapter 12 : Porphyrin and metalloporphyrin interactions with nucleic acids, by R. F. Pasternack and E. J. Gibbs
Chapter 13 : Selective DNA cleavage by metalloporphyrin derivatives, by G. Pratviel, J. Bernadou and B. Meunier
Chapter 14 : Synthetic metallopeptides as probes of protein-DNA interactions, by E. C. Long, P. D. Denney Eason and Q. Liang
Chapter 15 : Targeting of nucleic acids by iron complexes , by A. Draganescu and T. D. Tullius
Chapter 16 : Nucleic acid chemistry of the cuprous complexes of 1,10-phenantroline and derivatives, by D. S. Sigman, R. Landgraf, D. M. Perrin and L. Pearson
Chapter 17 : Specific DNA cleavage by manganese(II) complexes, by D. J. Gravert and J. H. Griffin
Chapter 18 : Nickel complexes as probes of guanine sites in nucleic acid folding, by C. J. Burroxs and S. E. Rokita
Chapter 19 : Hydrolytic cleavage of RNA catalyzed by metal ion complexes, by J. R. Morrow
Chapter 20 : RNA recognition and cleavage by iron(II)-bleomycin, by J. M. Battigello, M. Cui and B. J. Carter
Chapter 21 : Metallobleomycin-DNA interactions : Structures and reactions related to bleomycin-induced DNA dammage, by D. H. Petering, Q. Mao, W. Li, E. DeRose and W. E. Antholine

Chapter 1 expresses the methodological problems that are encountered by molecular modeling and delineates possible solutions to these problems. Several applications of molecular modeling to platinum-oligonucleotides complexes are reviewed.
Chapter 2 demonstrates that the $Zn^{II}$-cyclic complex and its functionalized derivatives are a new type of ideal receptor molecules that in neutral aqueous solution recognize and bind selectively and reversibly to thymine base and its homologs.

Chapter 3 presents the fundamental chemical and physiochemical properties of metallocene complexes that are relevant to their cytostatic activity.
Chapter 4 summarizes some results showing the active participation of the DNA double helix in transformation and/or removal of the adducts under physiological conditions.
Chapter 5 surveys literature data both on the basic chemistry of *trans*-$(NH_3)_2PtCl_2$ and its relatives and their reactions with DNA, oligonucleotides and model nucleobases.
Chapter 6 presents a survey of current data and draws some general conclusions on the interactions between metal ions and multiple stranded DNA.
Chapter 7 deals with the interaction of ruthenium complexes with DNA, a particular reference being given to the ongoing controversy about the modes of interaction of $[Ru(phen)_3]^{2+}$ and to recent studies with the complex $[Ru(phen)_2(DPPZ)]^{2+}$ which can truly be considered as an intercalator.
Chapter 8 studies the effects of some metal ions from the first transition series on the photodestruction of dyes intercalated in DNA. It investigates also the electron excitation energy transfer from AO to EB in DNA complexes.
Chapter 9 relates the development of new, photoactivated chemotherapeutic agents, specifically those involving covalent binding of the metal to the target biological substrate.
Chapter 10 describes approaches to obtain information on DNA structure and redox reactivity.
Chapter 11 focuses on studies from the autor's laboratories and presents some general chemical features that are emerging concerning the DNA wire.
Chapter 12 describes the interactions between porphyrins and different nucleic acid structures (duplex DNA, branched DNA, ...)
Chapter 13 describes two modes of activation for the DNA cleavage by regular metalloporphyrins: by light and by oxidants. A detailed mechanism is presented for manganese(III)*meso*-tetrakis-(4-N-methylpyridiniumyl)porphyrin activated by potassium persulfate.
Chapter 14 surveys the ongoing use of metallopeptides in the study of protein-DNA interactions including strategies for their synthesis and applications to biological problems.
Chapter 15 discusses the production in solution of the hydroxyl radical and its chemistry with nucleic acids a chemical probe wich can provide detailed information about nucleic acid structure.
Chapter 16 focuses on the binding specificity and reactivity of the 2:1 1,10-phenantroline-cuprous complex $[(OP)_2Cu^+]$, which exhibits a remarkable preference for different nucleic acid structures as well as for stressed DNA structures formed in enzymatic reactions.
Chapter 17 concerns DNA binding and cleaving properties of $[Salen\text{-}Mn(III)]^+$ and substituted derivatives.
In chapter 18 three nickel complexes have been studied for their role in promoting guanine-specific oxidation that is dependent on the exposure of the nucleobase to folded nucleic acids.
Chapter 19 describes the general mechanisms of hydrolytic cleavage by metal ions and some important considerations in ligand design, followed by a discussion of the effect of RNA structure and sequence on cleavage rates.
Chapter 20 demonstrates that the Fe(II)-BLM complex cleaves every major class of RNA and presents the therapeutic relevance of this cleavage.
Chapter 21 gives details of the cellular and chemical interactions between metallobleomycin and DNA focused on the mechanism of action of the drug to cause DNA damage.

In conclusion it can be stated that this high standard book is very helpful to every researcher interested by the influence of metal ions on nucleic acids structures and properties, and particularly for bioinorganic chemists.

